# Discussion of ‘Event history and topological data analysis’

**DOI:** 10.1093/biomet/asab023

**Published:** 2021-11-15

**Authors:** MOO K. CHUNG, HERNANDO OMBAO

**Affiliations:** Department of Biostatistics and Medical Informatics, University of Wisconsin-Madison, Medical Science Center 4725, 1300 University Avenue, Madison, Wisconsin 53706, U.S.A.; Statistics Program, King Abdullah University of Science and Technology (KAUST), Thuwal 23955, Saudi Arabia

## 1. Introduction

Although topological data analysis has been around for many decades with well-grounded theoretical development, it still suffers from numerous statistical and computational issues. For these reasons, it has not yet become a standard tool for data scientists. The authors point out the difficulty of directly applying existing statistical models to persistent homology due to the heterogeneous nature of topological features. The statistical development in topological data analysis in the last decade has been focused on making heterogeneous features into homogenous structured data by transformations or smoothing. Thus, the idea of applying survival analysis techniques to the birth and death process of topological features is very intriguing. The authors succeeded in elucidating the connection between event history methods and the lifetime of topological features, and the paper has stimulated many new interesting questions.

## 2. Trees in persistent homology

One of the most popular applications of persistent homology are on binary trees (Bendich et al., 2016; Li et al., 2017). Trees and graphs are 1-skeletons, which are Rips complexes consisting of only nodes and edges. Trees do not have 1-cycles and can be quantified using 0-cycles only. Other higher-order topological features are simply ignored. However, Garside et al. (2021) used somewhat inefficient filtrations in the 2D plane that increase the radius of circles from the root node or points along the tree. Such filtrations produce persistent diagrams that spread points in a 2D plane. Further, such an approach creates 1-cycles that may not really be needed in analysing trees. These types of persistent diagrams are difficult to analyse since the locations of the scatter points and the number of scatter points do not correspond across different persistent diagrams. For a 1-skeleton, there exists a more efficient 1D filtration called the graph filtration, which filters edge weights varying from −∞ to ∞ (Chung et al., 2019; Songdechakraiwut & Chung, 2020b).

Given a binary tree with node set *V* = {1, 2, *…*, *p*}, define a weighted tree T=(V,w) with the edge weight *w* = (*w*_*ij*_). The edge weight *w*_*ij*_ is given by the distance between nodes *i* and *j* if they are connected and 0 otherwise. Assume that the edge weights are all unique so that we can build the order statistics:

$$\underset{i,j}{\text{min }}w_{ij}=w_{\left(1\right)}<w_{\left(2\right)}<\cdots <w_{\left(p-1\right)}=\underset{i,j}{\text{max }}w_{ij}.$$

**Fig. 1.**
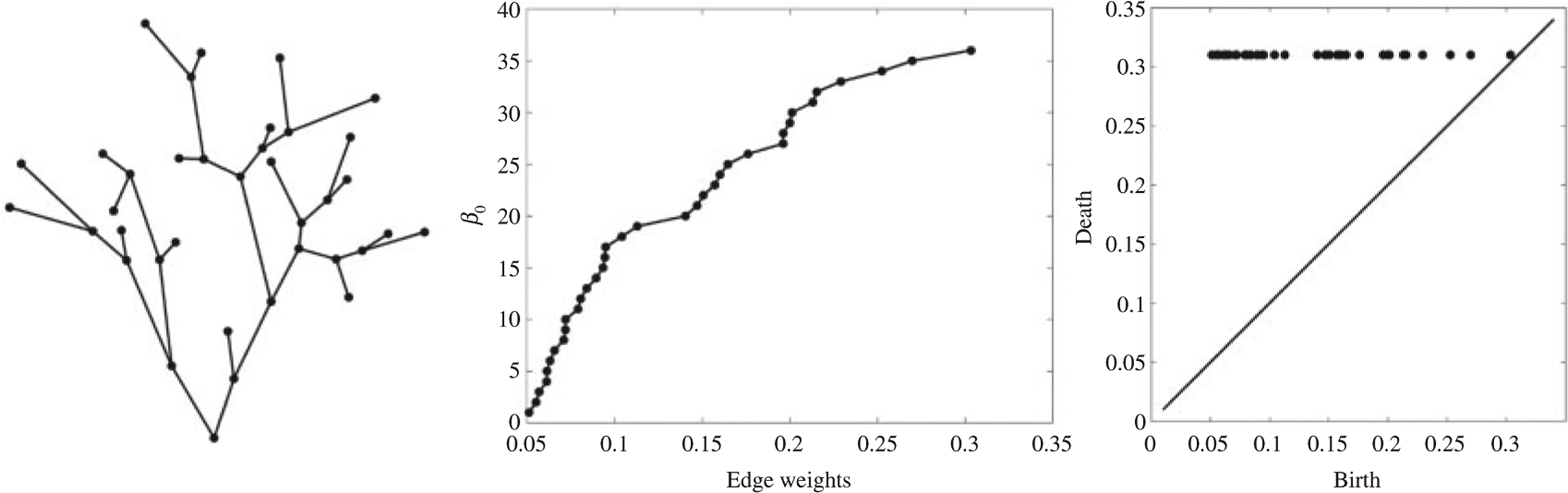
(a) Binary tree used in Garside et al. (2021). (b) *β*_0_-curve over graph filtration. The edge weights of the tree are used as the filtration values. (c) The points in the persistent diagram all lined up at *y* = 0.31.

Now threshold the weighted tree T at *ϵ*, which leads to the binary tree $T_{\epsilon }=\left(V,w_{\epsilon }\right)$ with edge weights *w*_*ϵ*_ = (*w*_*ϵ*, *ij*_), *w*_*ϵ*, *ij*_ = 1 if *w*_*ij*_ *> ϵ* and 0 otherwise. Finally, we obtain the graph filteration

$$T_{w_{\left(1\right)}}⊃T_{w_{\left(2\right)}}⊃\cdots ⊃T_{w_{\left(q-1\right)}},$$

which completely characterizes the topology of the original binary tree. Since $T_{\epsilon }$ is a collection of binary trees, there is no 1-cycle. Each time a new threshold is applied, the tree splits into two parts. Thus, the 0-th Betti number *β*_0_ is monotonically increasing over the filtration. In fact, $\beta_{0}\left(T_{w_{\left(i\right)}}\right)=i+2$ (Chung et al., 2019). None of the 0-cycles ever die once they are born. For convenience, we set the death value of 0-cycles to some fixed number *c > w*_(*q*−1)_. Then the persistence diagram of the graph filtration is simply (*w*_(1)_, *c*), (*w*_(2)_, *c*), …, (*w*_(*q*−1)_, *c*) forming 1D scatter points along the horizontal line *y* = *c*, and making various analysis and operations, including matching, significantly simplified (Songdechakraiwut & Chung, 2020b). Figure 1 illustrates the graph filtration and corresponding 1D scatter points in persistence diagrams on the binary tree used in Garside et al. (2021). In this example, *c* = 0.31 is arbitrarily picked to be larger than the maximum edge weight 0.3034.

A different graph filtration is also possible by making the edge weight to be the shortest distance from the root node. This filtration also carries the identical topological information. For general graphs beyond trees, there will be 1-cycles and *β*_1_ is monotonically decreasing over the graph filtration (Chung et al., 2019). Similarly, the persistence diagram is given as 1D scatter points along the vertical line (Songdechakraiwut & Chung, 2020b). Subsequently, statistical analysis on *β*_0_, *β*_1_ curves as well as their persistence diagram can be performed using existing tools in the order statistics.

## 3. Accumulating persistence

Garside et al. (2021) proposed modelling the birth and death of cycles as the observed data in event history analysis in a literal sense. Event history analysis has been widely used in diverse areas, including survival analysis in medicine and failure time analysis in engineering, and thus such an approach would open a new direction for research. From Garside et al. (2021), other event history approaches can be equally applicable to the birth and death of cycles. The Nelson–Aalen method and many other event history methods all bypass the problem of matching births and deaths across different subjects by accumulating events. In Garside et al. (2021), functions *N*_*x*_*(t)* and *Y (t)* accumulate the indicator variables for the events by summation. Such an approach usually yields the Nelson–Aalen plot type of monotone curves, which make the subsequent analysis stable and easy to perform. Although other authors did not make the connection to event history analysis, barcodes have often been accumulated into a summary statistic. In Biscio & Møller (2019), the accumulated persistence function, which simply sums the length of barcodes, is proposed for brain artery trees. In Songdechakraiwut & Chung (2020a), barcodes are also accumulated for time series data. In graph filtration, the accumulating barcode is equivalent to computing the area under the Betti curves. It would be of interest to investigate various accumulation strategies beyond the Nelson–Aalen estimator. Garside et al. (2021) have opened a new research direction.

## 4. Lack of localization

**Fig. 2.**
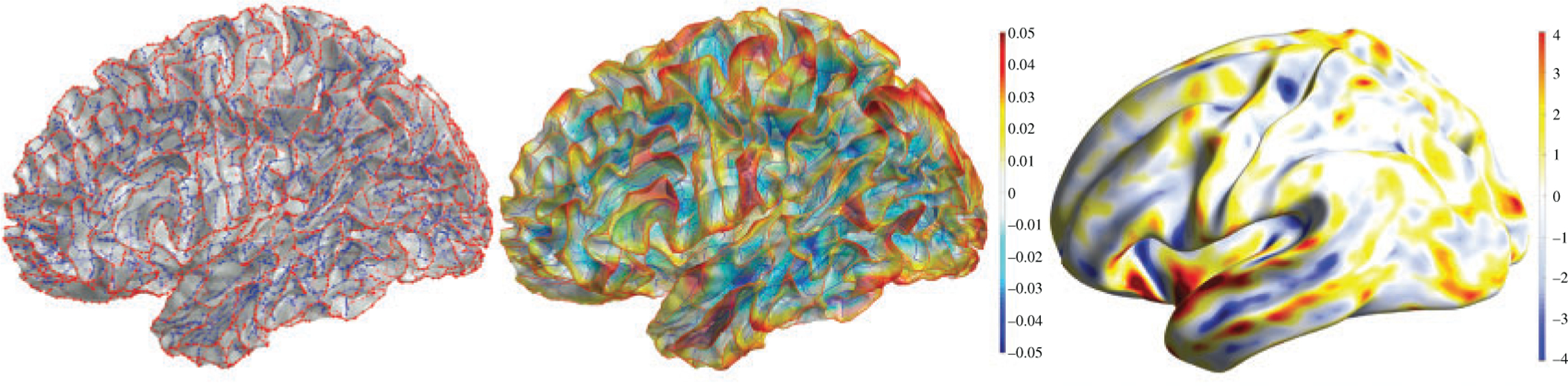
(a) Sulcal (blue) and gyral (red) trees of brain cortical surface mesh (Huang et al., 2020). (b) Diffusion of sulcal trees (value −1) and gyral trees (value +1). (c) Two-sample *t*-statistic on 268 females and 176 males localizing the sexual diffemorphism in the temporal lobes.

The approach in Garside et al. (2021) succeeded in differentiating the vascular tree patterns between healthy and diabetic retinopathy patients. However, their method does not clearly identify the location of the difference within the vascular tree. In related problems in medical diagnostics, it is important to determine the topological differences. However, a more important question is localizing the source of differences. Since there is no one-to-one map between the transformed topological features and the original data space, it is often not possible to localize the signals. In our opinion, this has been the biggest limitation of the topological data analysis methods in biomedical data. Thus, we believe that development of topological data analysis methods should be towards this important, though very difficult, question. Compared to topological data analysis methods, geometric methods are more adept at detecting localized signals in trees. Figure 2 displays the sucal and gyral trees obtained from brain surface meshes (Huang et al., 2020). Trees are treated as a heat source with value +1 on gyral trees and a heat sink with value −1 on sulcal trees. Then isotropic diffusion is performed to produce the smooth map of sulcal and gyral trees. The major advantage of this approach is that such maps can be easily compared across different subjects. In Huang et al. (2020), a two-sample *t*-statistic is calculated at each mesh vertex and is used in localizing the sex difference, 268 females, 176 males, near the temporal lobes of the brain. Such localized signal detection is not possible with many existing topological data analysis methods. Persistent homology features are, by definition, global summary measures, and they might be more useful for tasks that do not involve identifying the source of signal differences. Thus, they might be more useful in discrete decision-making tasks such as clustering and classification. In fact, topological data analysis has begun to be more useful in deep learning (Chen et al., 2019) and in identifying shared common features in time series (Wang et al., 2015, 2018).

## 5. Consistency versus stability

Although topological data analysis has been applied in various fields, it still lacks the rigorous foundation for statistical inference. Most topological data analysis features enjoy the stability property that shows that the distance *d* between two topological features is bounded by some known well-behaved distances (Cohen-Steiner et al., 2007; Adams et al., 2017). However, to build the proper statistical framework we need statistical consistency (Bubenik et al., 2010). Given the average topological feature *T*_*n*_ over *n* samples, we need the following topological version of consistency that shows the convergence to true population signal *T* in probability: lim_*n*→∞_ *P*{*d*(*T*_*n*_, *T*) *> ϵ*} = 0 for all *ϵ >* 0. Since most topological features such as persistence diagrams do not form a vector space, it is not immediately obvious how to even define the expectation and variance of topological features *T*_*n*_. Such consistency guarantees the convergence of statistical results for a sufficient sample size. Existing stability results are mostly on the stability of topological data analysis features, but not about the consistency of the test statistics on such features. Additional investigations are needed to establish the consistency of statistics built on top of topological data analysis features. The consistency of the Nelson–Aaelen estimator and other accumulation-based survival functions is well established (Andersen et al., 2012). Thus, the use of survival analysis methods will automatically bring the needed consistency results.
